# Acute Methaemoglobinaemia as an Overlooked Cause of Acute Dyspnoea in a Patient With Chronic Obstructive Pulmonary Disorder in the Emergency Department

**DOI:** 10.7759/cureus.92259

**Published:** 2025-09-14

**Authors:** Laurat Zoaka, Zahra Al-Zoubeidi, Shafa Khokhar, Tarek Abdelaziz, Aloaye Foy-Yamah

**Affiliations:** 1 Respiratory Medicine, Blackpool Victoria Hospital, Blackpool Teaching Hospitals NHS Trust, Blackpool, GBR; 2 Acute Medicine, Blackpool Victoria Hospital, Blackpool Teaching Hospitals NHS Trust, Blackpool, GBR

**Keywords:** alkyl nitrates, chronic obstructive pulmonary disease (copd), copd, dyspnoea, hypoxia, methaemoglobin, methaemoglobinaemia, recreational drugs

## Abstract

Methaemoglobinaemia is a condition characterised by excessive methaemoglobin (MetHb), which is an oxidised form of haemoglobin in the blood. MetHb is unable to bind oxygen, subsequently leading to hypoxia. Methaemoglobinaemia can either be congenital or acquired, often caused by exposure to oxidising agents like alkyl nitrates, commonly found in illicit drugs.

This case report is of a 45-year-old man with chronic obstructive pulmonary disorder (COPD), who developed methaemoglobinaemia as a result of recreational use of alkyl nitrates. This patient presented with worsening dyspnoea and blood gases showing high MetHb, which was initially overlooked. His leukocyte and neutrophil levels were found to be elevated. The initial impression was an infective exacerbation of COPD (IECOPD). The suspicion of acute methaemoglobinaemia was made only once he disclosed his alkyl nitrate usage. He was then transferred to the high dependency unit and received hyperbaric oxygen and ascorbic acid, alongside treatment for IECOPD.

Significant learning includes the importance of considering methaemoglobinaemia in dyspnoeic patients, consideration of methaemoglobinaemia as a differential diagnosis to IECOPD, and the importance of obtaining a thorough history of recreational substance usage in anyone with methaemoglobinaemia.

## Introduction

Methaemoglobinaemia is a haematological condition characterised by the presence of excessive methaemoglobin (MetHb) in blood. This is an oxidised form of haemoglobin with no oxygen-binding capacity. MetHb is usually neutralised in healthy individuals, leaving only trace amounts of ≤1% to-2%. Levels beyond 10% can cause hypoxia depending on the patient’s baseline haemoglobin level [1,2]. Methaemoglobinaemia can be congenital or acquired. The former is due to a deficiency in enzymes such as cytochrome B5 reductase or because of unusual haemoglobin variants (HbM, HbH), which are irreducible by normal enzyme systems [2]. Acquired methaemoglobinaemia can be caused by exogenous oxidising agents like local anaesthetics or chemical agents. This is especially the case in infants and pregnant women, as they have a higher predisposition to accumulate MetHb owing to their physiological inadequacy of enzyme stores in the immature or gravid states. There is also an increased risk in areas with nitrate-rich water supplies [2].

Rising MetHb levels lead to hypoxia, resulting in cyanosis, anxiety, dyspnoea, headaches, dizziness, nausea, tachycardia, and tachypnoea. Eventually, it can lead to cardiac arrhythmias, altered mental state, seizures, comas, and ultimately death. Signs of methaemoglobinaemia include chocolate-coloured blood and central cyanosis [1,2]. Management requires removing the causative agent and considering treatment with methylene blue or ascorbic acid if contraindicated, as well as delivering high-flow oxygen to increase oxygen delivery to tissues and help the natural degeneration of MetHb [2]. Methylene blue is contraindicated in conditions such as renal impairment, G6PD deficiency, and Heinz body haemolytic anaemia. In these cases, urgent transfusion with O-negative blood or exchange transfusion should be considered [2].

Alkyl nitrates, also known as poppers, rush, liquid gold, or TNT, are recreational vasodilatory inhalants that cause relaxation of smooth muscles, inducing a euphoric ‘rush’, heightened skin sensitivity, and libido [3]. They are easily available for purchase. This case features a patient with a background of chronic obstructive pulmonary disease (COPD) who took alkyl nitrates recreationally, resulting in methaemoglobinaemia, and the management thereof. It is unique in that it focuses on alkyl nitrate use in COPD patients and the importance of creating a non-judgemental environment for patients to disclose their recreational drug use.

## Case presentation

A 45-year-old Caucasian male with COPD presented to the emergency department (ED) complaining of progressive, worsening shortness of breath over a few days. He had an oxygen saturation of 82% on room air (fraction of inspired oxygen (FiO₂) 21%), and central cyanosis was noted in the community by his general practice nurse. He had recently developed a productive cough with yellow phlegm. He denied chest pain, palpitations, and haemoptysis. His background included COPD, Raynaud’s phenomenon, depression, and penicillin allergy. He lived with his wife and was independently mobile. His drug history included mirtazapine, procyclidine, hyoscine, aripiprazole and formoterol. He reported a smoking history of 20 cigarettes per day for around 20 years.

Observations showed saturations <91% on room air, a heart rate of 91 beats per minute (bpm), and blood pressure of 129/70 mmHg, and the patient was alert and afebrile. Respiratory examination revealed normal chest expansion with bilaterally equal and reduced breath sounds, along with audible crackles over the right lung and scattered wheezes. Cardiovascular examination was unremarkable. There were no peripheral signs of venous thromboembolism or cellulitis. 

**Figure 1:**
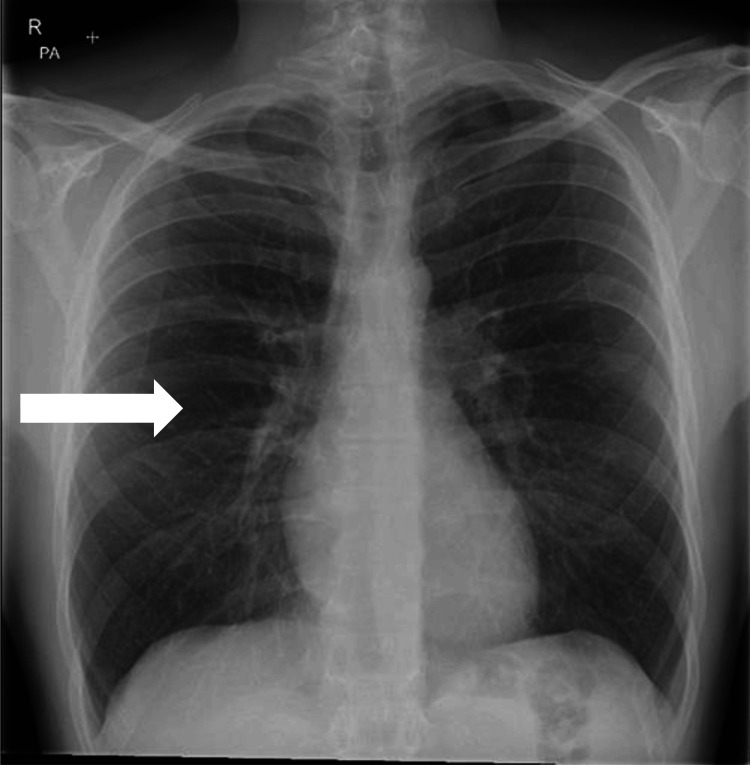
Chest X-ray of Patient Taken on Admission Showing Lung Hyperinflation This chest X-ray shows hyperinflation. There are more than six ribs visible anteriorly. The arrow indicates widened intercostal spaces, also typical of lung hyperinflation in COPD. No acute pathology can be appreciated. COPD: chronic obstructive pulmonary disorder

The biochemical and haematological profile showed leucocytosis (10.9 x 109/L) with mild neutrophilia. A marked MetHb elevation of 10.6% was evident on his arterial blood gas (ABG) (Table 1). There was lung hyperinflation on the chest X-ray, but no acute changes (Figure 1). His electrocardiogram (ECG) showed a normal sinus rhythm.

**Table 1:** Serial Blood Gases of the Patient Taken On the First Day of Presentation, Before, and After Oxygen Therapy

Serial blood gases	Morning (AM)	Evening (PM)	Normal values
Methaemoglobin (MetHb, %)	10.6	8.4	0.0-3.0
pH	7.38	7.43	7.35-7.45
Partial pressure of carbon dioxide (pCO_2_, kPa)	6.7	4.6	4.5-6.0
Bicarbonate (HCO3, mmol/L)	25.8	24	21.0-28.0
Base Excess (BE, mmol/L)	3.5	-0.7	-2.0 to 3.0

Management primarily focused on treatment of an infective exacerbation of COPD (IECOPD) with oxygen therapy, oral doxycycline 200mg once daily, oral prednisolone 30mg once daily, and salbutamol and ipratropium nebulisers. The methaemoglobinaemia was initially overlooked in the ED; however, it improved with the administration of oxygen over 12 hours (starting on a 15L non-rebreather mask, then reduced to 6L via nasal cannula). 

In the beginning, the patient denied any exposure to methaemoglobinaemia-inducing substances, but after explaining the relevance of this, he disclosed his recreational use of alkyl nitrates (colloquially known as poppers, rush, liquid gold, or TNT). As mentioned previously, acquired methaemoglobinaemia can be caused by exogenous oxidising agents (Table 2) [1]. The treatment for methaemoglobinaemia is methylene blue, which works by reducing MetHb to haemoglobin [1]. However, by this time, the patient no longer qualified for intravenous methylene blue treatment, as his MetHb had fallen to 8.4% with simple oxygen therapy. He was immediately transferred to a high-dependency unit where he received treatment with hyperbaric oxygen, 400 mg ascorbic acid over 24 hours, and further IECOPD management. This facilitated a rapid recovery over 48 hours with resolution of the IECOPD and acute methaemoglobinaemia, as evidenced by the improving clinical picture and serial blood gases. The patient tolerated and adhered to the management, as seen by the successful treatment of his methaemoglobinaemia with no adverse or unanticipated events. Subsequently, he was discharged home with follow-up with respiratory nurses in the community for long-term management of his COPD. No follow-up diagnostics were arranged by the hospital team. 

**Table 2:** Exogenous Substances Which Can Cause Acquired Methaemoglobinaemia Source: [1]

Drugs /Chemical Agents
Benzocaine
Lidocaine
Prilocaine
Articaine
Dapsone
Trimethoprim
Sulphonamide
Metoclopramide
Aniline dyes
Chlorates
Bromates
Nitrites
Nitrates from natural sources
Nitrates from recreational drugs (poppers etc.)

This patient’s management included hyperbaric oxygen, which rapidly enhances oxygen delivery to the tissues and mitigates the hypoxic effects of MetHb [4]. This is especially the case in severe or refractory cases of methaemoglobinaemia. Although hyperbaric oxygen does not directly reduce MetHb levels, it increases the amount of dissolved oxygen in the blood, thereby compensating for the impaired oxygen-carrying capacity until definitive measures such as methylene blue can be administered. It is also effective in patients who do not respond to standard treatment or have contraindications to methylene blue. Some studies have shown that ascorbic acid (vitamin C) can be used intravenously in the treatment of methaemoglobinaemia [2,5]. This reduces MetHb to functional haemoglobin. Ascorbic acid is well-tolerated and can be used in patients with contraindications to methylene blue. Additionally, it is readily available and inexpensive. It does, however, act more slowly than alternative treatments, and it has limited efficacy in severe or acute methaemoglobinaemia. Moreover, caution is advised in patients with renal insufficiency, as high-dose ascorbic acid use can cause increased urinary excretion of oxalate (hyperoxaluria), which can cause renal failure [5]. Although the final blood gas measurement prior to discharge was unavailable, the discharge summary documented a methaemoglobin level of 2.4%, confirming resolution of the acute episode. This represents a limitation of the case, as serial gasometric monitoring would have allowed for a more objective assessment of treatment response.

## Discussion

Learning points

Methaemoglobinaemia should be considered in hypoxic COPD patients when symptoms are disproportionate to findings. Methaemoglobinaemia can mimic IECOPD, presenting with symptoms like dyspnoea, cyanosis, and fatigue. Alternative diagnoses must be explored when oxygen saturation is low despite oxygen therapy, and imaging or infection markers don’t fully support IECOPD. Anchoring bias can delay diagnosis and treatment. 

MetHb levels must always be checked when interpreting arterial blood gases in unexplained hypoxia. Ascertaining the MetHb value (which is often overlooked) should be a vital part of ABG analysis, especially in COPD patients [6]. A key diagnostic clue is a “saturation gap”: where pulse oximetry (SpO₂) reads significantly lower than oxygen saturation (SaO₂) calculated from ABG [7]. Pulse oximetry is unreliable in methaemoglobinaemia due to altered light absorption [7]. Co-oximetry, or direct MetHb measurement, although not used here, has been shown to be an important diagnostic tool in many cases [8,9]. 

Methaemoglobinaemia must be treated urgently as an acquired condition until proven otherwise. In the acute setting, acquired methaemoglobinaemia is more likely than congenital causes. Prompt identification and treatment are essential to prevent complications. Oxygen therapy should be started immediately, and definitive treatment (methylene blue) should be guided by severity and contraindications. 

A thorough drug history, including recreational substances, must be taken in any case of unexplained hypoxia. Recreational drug use is a sensitive topic, often omitted by patients unless directly and compassionately explored. Alkyl nitrates (poppers) are easily accessible and not always recognised as harmful. Clear, empathetic communication explaining the relevance of the information increases the likelihood of disclosure, which will improve care. 

Patients with COPD, anaemia, or congestive cardiac failure are more susceptible to severe effects of methaemoglobinaemia at lower MetHb levels. In these patients, even mild elevations in MetHb can tip them into symptomatic hypoxia due to impaired oxygen delivery [8]. A lower threshold for treatment should be considered in patients with pre-existing hypoxaemic or circulatory conditions. 

Hyperbaric oxygen and ascorbic acid are effective alternatives when methylene blue is contraindicated [10]. While methylene blue remains first-line, alternatives like hyperbaric oxygen and high-dose vitamin C are viable, especially in G6PD deficiency or renal impairment. Ascorbic acid acts more slowly but is accessible, inexpensive, and well-tolerated. 

The true prevalence of methaemoglobinaemia may be underestimated. Due to symptom overlap with common respiratory conditions, reliance on pulse oximetry, and lack of routine MetHb measurement, many cases may go unrecognised. Increased awareness, especially in the context of recreational drug use, is essential for earlier diagnosis and improved outcomes. 

It is imperative to treat all conditions contributing to the patient’s presentation alongside the methaemoglobinaemia. Whilst timely management of acute methaemoglobinemia is crucial, it is equally important not to overlook or leave untreated any exacerbation of an existing chronic hypoxemic condition. In this case, the patient presented with both acute methaemoglobinemia and IECOPD, both of which required management for full recovery. 

Strengths and limitations

This case study describes a rare presentation with high-impact learning points. Whilst methaemoglobinaemia is rare, it can be fatal, so timely recognition and management are crucial. Methaemoglobinaemia due to alkyl nitrate usage in COPD is rare, and recognition is often missed due to symptom overlap with more common respiratory conditions. It is important to avoid anchoring to one diagnosis, which can lead to incorrect management. Therefore, this publication highlights both the significance of this case as a learning tool and the importance of being vigilant in further cases. 

Another strength is that this case follows the patient from presentation through his hospital admission to resolution of his methaemoglobinaemia. This case also highlights diagnostic bias and the importance of taking a thorough history. It also focuses on providing a non-judgemental space for patients to share their recreational drug history, emphasising ethical practice and holistic care, an important aspect of good medical care. This will hopefully empower clinicians to reflect on communication and their own stigmas. This study also discusses alternative therapies to methylene blue, such as high-dose ascorbic acid and hyperbaric oxygen, broadening understanding of therapeutic options in methaemoglobinaemia management, which can have further applications in cases where methylene blue is contraindicated. This study used ABG as a diagnostic tool for methaemoglobinaemia [6]. It did not use co-oximetry, despite this being the gold standard for the detection of methaemoglobinaemia [8,9]. This was largely due to resource availability. This is something that can be considered in future cases. 

This patient was not signposted to substance misuse services, which may have been useful considering his recreational drug use. Furthermore, this study does not explore social determinants of why people use substances like poppers, the stigma of this, or whether this impacts access to care. This is not within the scope of this publication, but can be explored further in further studies. One other limitation of this case is the incomplete long-term follow-up. This patient was followed up in the community, and he reported feeling well in himself, reporting a good quality of life and no complications from his admission. Unfortunately, he could not be followed up with further, as he passed away. It is unknown if this was related to alkyl nitrate usage. We are, therefore, unable to draw conclusions about long-term prognosis or recurrence risk and cannot comment on behavioural change or patient education. 

## Conclusions

In conclusion, this case report features a patient with COPD presenting with methaemoglobinaemia due to alkyl nitrate usage and its management. It is unique in that it focuses on alkyl nitrate use in COPD patients and the importance of taking an accurate recreational drug history in a non-judgemental environment. This is imperative in methaemoglobinaemia management, as it can be masked by IECOPD, given their similar clinical presentations. Finally, patients with COPD and other chronic hypoxic conditions are more likely to become symptomatic at lower concentrations of MetHb. Hopefully, this case will help emphasise the clinical significance of methaemoglobinaemia as a differential aetiology for dyspnoea in the acute setting, leading to more efficient management and better patient outcomes.
